# Early Imaging Biomarker of Myocardial Glucose Adaptations in High-Fat-Diet-Induced Insulin Resistance Model by Using ^18^F-FDG PET and [U-^13^C]glucose Nuclear Magnetic Resonance Tracer

**DOI:** 10.1155/2018/8751267

**Published:** 2018-07-12

**Authors:** Yi-Hsiu Chung, Kuan-Ying Lu, Shao-Chieh Chiu, Chi-Jen Lo, Li-Man Hung, Jiung-Pang Huang, Mei-Ling Cheng, Chao-Hung Wang, Cheng-Kun Tsai, Yu-Chun Lin, Shang-Hung Chang, Gigin Lin

**Affiliations:** ^1^Center for Advanced Molecular Imaging and Translation (CAMIT), Linkou Chang Gung Memorial Hospital, Taoyuan, Taiwan; ^2^Department of Medical Imaging and Intervention, Imaging Core Lab, Institute for Radiological Research, Linkou Chang Gung Memorial Hospital and Chang Gung University, Taoyuan, Taiwan; ^3^Clinical Metabolomics Core Laboratory, Linkou Chang Gung Memorial Hospital, Taoyuan, Taiwan; ^4^Metabolomics Core Laboratory, Healthy Aging Research Center, Chang Gung University, Taoyuan, Taiwan; ^5^Department and Graduate Institute of Biomedical Sciences, College of Medicine, Chang Gung University, Taoyuan, Taiwan; ^6^Healthy Aging Research Center, Chang Gung University, Taoyuan, Taiwan; ^7^Kidney Research Center, Linkou Chang Gung Memorial Hospital, Taoyuan, Taiwan; ^8^Heart Failure Center, Division of Cardiology, Department of Internal Medicine, Chang Gung Memorial Hospital, Keelung, Taiwan

## Abstract

**Background:**

High-fat diet (HFD) induces systemic insulin resistance leading to myocardial dysfunction. We aim to characterize the early adaptations of myocardial glucose utility to HFD-induced insulin resistance.

**Methods:**

Male Sprague–Dawley rats were assigned into two groups, fed a regular chow diet or HFD ad libitum for 10 weeks. We used *in vivo* imaging of cardiac magnetic resonance (CMR), ^18^F-FDG PET, and ex vivo nuclear magnetic resonance (NMR) metabolomic analysis for the carbon-13-labeled glucose ([U-^13^C]Glc) perfused myocardium.

**Results:**

As compared with controls, HFD rats had a higher ejection fraction and a smaller left ventricular end-systolic volume (*P* < 0.05), with SUV_max_ of myocardium on ^18^F-FDG PET significantly increased in 4 weeks (*P* < 0.005). The [U-^13^C]Glc probed the increased glucose uptake being metabolized into pyruvate and acetyl-CoA, undergoing oxidative phosphorylation via the tricarboxylic acid (TCA) cycle, and then synthesized into glutamic acid and glutamine, associated with overexpressed LC3B (*P* < 0.05).

**Conclusions:**

HFD-induced IR associated with increased glucose utility undergoing oxidative phosphorylation via the TCA cycle in the myocardium is supported by overexpression of glucose transporter, acetyl-CoA synthase. Noninvasive imaging biomarker has potentials in detecting the metabolic perturbations prior to the decline of the left ventricular function.

## 1. Introduction

Diabetes mellitus (DM) is a pandemic metabolic problem characterized by hyperglycemia due to defects in secretion or action of insulin, namely, type 1 or type 2 diabetes mellitus (T2DM), respectively. In 2015, 415 million people had DM in the world, and the number will grow to 522 million in 2030 with the conservative estimation [1, 2]. High-fat diet (HFD) is one leading cause of T2DM [3, 4], causing resistance to insulin in multiple organs [5]. The relatively subtle metabolic changes, such as moderate hyperglycemia and increased plasma triglyceride (TG) levels, can gradually lead to structural changes and heart failure [6, 7]. Some previous reports have shown that the myocardium of humans and animals with T2DM might switch energy production from glucose utilization to fatty acid oxidation [8–10], which may play a critical role in the pathogenesis of T2DM. The presence of hyperinsulinemia, obesity, and impaired glucose tolerance in the rats with HFD has been characterized as the prediabetic T2DM rat model in the literature [11]. The Randle cycle (glucose fatty-acid cycle), a metabolic process involving the switch between glucose and fatty acids for substrates [12], is theorized to play a role in explaining T2DM and IR [13, 14]. Although the energy balance from differing macronutrient composition is equal, the glucose and fat balances that contribute to the overall energy balance are supposed to change reciprocally with dietary composition [15]. Monitoring the metabolic switch in myocardium would help in early detection and treatment of the myocardial changes in T2DM.

Cardiac magnetic resonance (CMR) imaging is a noninvasive assessment of both ischemic and nonischemic heart disease and heart failure [16]. CMR has become the gold standard for evaluating the left ventricular (LV) function, providing objective structural and detailed functional readouts [17–19]. However, the measurement of myocardial metabolism remains a challenge for CMR. ^18^F-fluorodeoxyglucose (^18^F-FDG) is a glucose analog which has been extensively used in positron emission tomography (PET) in clinical oncological diagnosis and assessment of the treatment response [20]. ^18^FDG is taken up by cells and trapped in the cytoplasm as ^18^F-FDG-6-phosphate by hexokinase once it enters into cells through glucose transporters (Glut) [21]. Normal myocardium also actively uptakes ^18^F-FDG due to high expression of hexokinase 2 [22, 23], providing the opportunity of using ^18^F-FDG to probe the first command step of myocardium glucose utilization by the *in vivo* noninvasive micro-PET study. The Langendorff system perfused with carbon-13-labeled glucose ([U-^13^C]Glc) can potentially probe the downstream metabolic activities further to ^18^F-FDG-6-phosphate in the myocardium [5]. High-resolution nuclear magnetic resonance (NMR) technology can identify the following metabolic products of [U-^13^C]Glc to precisely characterize the myocardial glucose utilization in the HFD-induced insulin resistance (IR). However, noninvasive detection of early myocardial changes of the prediabetic T2DM rat model has not been investigated yet.

We aim to characterize the metabolic changes associated with myocardial glucose utilization in a rat model of HFD-induced IR as an early diabetic onset model using the noninvasive imaging of cardiac MRI, ^18^F-FDG micro-PET, and [U-^13^C]Glc NMR corroboration.

## 2. Methods

### 2.1. Animal Model and Experiment Design

**Figure 1:**
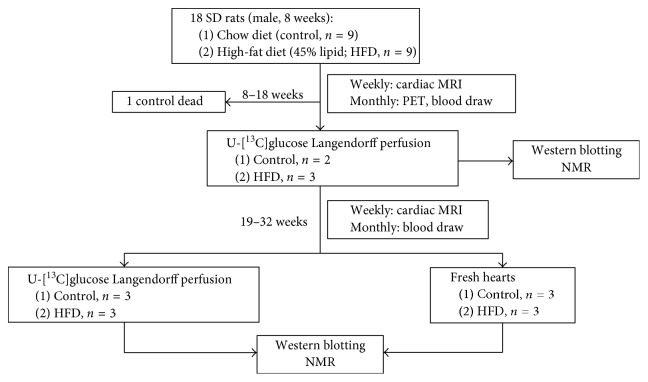
Flow chart of animal experiments. The age and number of animals used in each experiment are displayed. Rats were divided into two groups by the high-fat diet and regular chow diet, following the biochemical assessment, *in vivo* imaging study, Langendorff perfusion NMR, and western blotting.

We purchased male Sprague–Dawley (SD) rats (age 6 weeks) from BioLASCO Taiwan Co., Ltd. (Taipei, Taiwan). The SD rats were maintained in a climate-controlled facility on a 12 h light/12 h dark cycle with access to water and food ad libitum. All experimental procedures were conducted according to the Guide for the Care and Use of Laboratory Animals and were approved by the Institutional Animal Care and Use Committee of Chang Gung University and Chang Gung Memorial Hospital, Taiwan (IACUC: CGU13-051). After two weeks of acclimation, the SD rats were randomly assigned to 2 groups, fed a regular chow diet (control, C; *n*=9) or high-fat diet (HFD; *n*=9) ad libitum for 10 weeks. As our previous research, the regular chow diet consisted of 23.5% protein, 5.1% fat, and 50.3% carbohydrates (g %, LabDiet® 5001, St. Louis, MO); the HFD consisted of 24% protein, 24% fat, and 41% carbohydrates (g %, Research Diets D12451I, New Brunswick, NJ) [24]. We recorded their body weight and food intake weekly. In the SD rats (C, *n*=9; HFD, *n*=9), blood from the tail vein was withdrawn under fasting status for insulin-resistant testing. The insulin level was determined manually through Ultrasensitive Mouse Insulin ELISA Kit (Crystal Chem Inc., IL, USA). Briefly, a 5 *μ*l aliquot of plasma was applied in the ELISA kit. Then, the OD excitation wavelength 450 nm/emission wavelength 630 nm was used in detection. The sensitivity of Ultrasensitive Mouse Insulin ELISA Kit is 0.05 ng/mL. The glucose level in whole blood was measured by ONETOUCH® ULTRA® (Johnson & Johnson) with a drop test in a test strip. The biochemistry indexes, such as triacylglycerol (TG), total cholesterol (T-CHO), and high-density lipoprotein (HDL-C), were detected by Fujifilm NX500i (Japan) with 10 *μ*l of plasma following the ELISA kit protocol. The measurement ranges of glucose, TG, T-CHO, and HDL-C are 19.62–600 mg/dL, 10–500 mg/dL, 50–450 mg/dL, and 10–110 mg/dL, respectively. Homeostasis model assessment-insulin resistance (HOMA-IR) was calculated using HOMA2 calculator [25] and has been reported as a useful tool for diagnosing insulin resistance [26]. We carried out cardiac MRI weekly for 10 weeks (Control, *n*=9; HFD, *n*=9) and ^18^F-FDG PET on week 1, week 4, and week 7 (Control, *n*=3; HFD, *n*=3) after HFD feeding. We further analyzed the heart by using the [U-^13^C]Glc Langendorff perfusion study, NMR, and western blotting at 18 weeks of age (Control, *n*=2; HFD, *n*=3). In comparison to the early phase of pre-DM status, we analyzed the hearts with and without Langendorff perfusion by NMR and western blotting at 32 weeks of age (Control, *n*=3; HFD, *n*=3), as detailed below. The flow chart of the performing animal and tissue experiments was summarized in Figure 1.

### 2.2. CMR

CMR was performed with ECG and respiratory gating on the ClinScan 7T MRI (Bruker BioSpin, Germany). Short-axis CINE with the bright-blood technique was chosen to capture myocardial wall motion and thickness between end-systole and end-diastole phases. Specific parameters of CINE using FLASH sequence are TR/TE 12/1.67 ms, slice thickness 1 mm, three averages, flip angle 15°, the field of view 45 × 45 mm^2^, and 144 × 192 matrix, which gives rise to in-plane resolution 0.312 × 0.234 mm^2^. Such CINE scans were acquired sequentially and evenly from the base to the apex, yet the slice numbers to cover the whole heart and the corresponding acquisition time per incidence slightly varied among different animals and ECG stability. All CINE frames were quantitatively analyzed using QMass (Medis, the Netherlands), with manual delineation of the myocardium for left ventricular volume, ejection fraction, wall motion, and wall thickness.

### 2.3. Micro-PET

The SD rats were imaged using an InveonTM system (Siemens Medical Solutions Inc., Malvern, PA, USA) at Chang Gung Memorial Hospital, Taiwan. All rats were fasted for 12 hours before micro-PET but allowed ad libitum to access water. Although the blood glucose level of each rat was not measured before the micro-PET study, in the previous reports, there is no significant difference in glucose levels of the control and HFD rats with 12-hour fasting [27]. The rats underwent a 30 min image acquisition in the prone position 90 min after receiving 22.49 ± 0.17 (standard deviation, SD) MBq of ^18^F-FDG via tail vein injection. The rats were anesthetized with 3% isoflurane, and the heart of the rat was positioned near the center of the field of view where the spatial resolution is approximately 1.2 mm. The regions of interest (ROIs) of the myocardium were analyzed in the PET images manually. The ^18^F-FDG uptakes of the myocardium were expressed as the maximum standardized uptake value (SUV_max_) [28]. All image analyses were conducted by using PMOD version 3.2 image analysis software (PMOD Technologies Ltd., Zurich, Switzerland).

### 2.4. Isolated Heart Perfusion

Rats were anesthetized with sodium pentobarbitone (50 mg/kg, intraperitoneal injection, IP) and given heparin (300 units/kg, IP), following a cervical dislocation. Immediately, hearts were isolated in ice-cold Krebs–Henseleit buffer [21], cannulated via the aorta, and perfused in Langendorff mode at a constant perfusion pressure of 100 mmHg at 37°C [29]. Hearts were perfused with KH buffer (137.0 mM NaCl, 5.4 mM KCl, 1.22 mM MgSO_4_, 1.8 mM CaCl_2_, 1.2 mM KH_2_PO_4_, 11.0 mM dextrose, and 6.0 mM HEPES, pH 7.4), gassed with 95% O_2_ and 5% CO_2_ in steady perfusion for 10 min. Then, the dextrose in KH buffer was replaced with [U-^13^C]Glc, following 2 min perfusion. The hearts were removed from the Langendorff perfusion system after completing the perfusion, and each compartment of the heart was separated at the saline cooling bath. Each compartment of the hearts was wrapped in an aluminum foil and stored in liquid nitrogen for further usage.

### 2.5. NMR Metabolomic Study and Data Analysis

NMR spectra were acquired using a Bruker Avance III HD 600 MHz spectrometer operating at 600.13 MHz and equipped with a TXI CryoProbe at 300 K. Two types of ^1^H NMR spectra were acquired: NOESY and Carr–Purcell–Meiboom–Gill (CPMG) pulse sequence. The analyzed aqueous metabolites were glucose, lactate, acetate, glutamine, and glutamate; the analyzed lipophilic metabolite was a long-chain lipid component (CH_2_)*n*. Using the Bruker TopSpin 3.2 software and Chenomx Profiler 8.0, we integrated the NMR spectra for the HFD and control groups. The ratio of the metabolic concentrations of HFD and control groups at 18 and 32 weeks was determined, respectively. The metabolic levels of the NMR spectra were computed by the internal reference of sodium 3-trimethylsilyl-2,2,3,3-tetradeuteropropionate (TSP) in the aqueous phase and of tetramethylsilane (TMS) in the lipophilic phase. The sum of ^13^C resonances at the 4th and 5th positions of glutamate was compared with the sum of ^13^C resonances at the 1st, 2nd, and 3rd positions (Glu45/Glu123 ratio).

### 2.6. Western Blotting

Myocardium lysates were analyzed by western blotting, as described previously [30]. The myocardium lysate protein was transferred onto Immun-Blot PVDF membranes (Bio-Rad, USA). PVDF membranes were incubated with Glut4 (1F8) Mouse mAb, AceCS1 (D19C6) antibody (Cell Signaling, MA, USA), caspase-3 antibody (Cell Signaling, MA, USA), PARP antibody (Cell Signaling, MA, USA), LC3B antibody (Cell Signaling, MA, USA), and *β*-actin (as a loading control; Sigma-Aldrich, Israel). The membranes were then incubated with the anti-rabbit or anti-mouse secondary antibody (Sigma-Aldrich, Israel). Specific binding antibody-target protein interactions were rinsed with ECL (BioRad, USA) and detected under the chemiluminescence system (BioSpectrum UVP, CA, USA). Each blotting band was normalized with its loading control and quantitated utilizing ImageJ 1.49t.

### 2.7. Relative Gene Expression Analysis for Slc2a4 (Glut4) and Acss2 (AceCS1)

First, total RNA of each sample was extracted using AurumTM Total RNA Fatty and Fibrous Tissue Kit (Bio-Rad, USA) and was quantified by NanoDrop 1000 Spectrophotometer (Thermo Scientific, USA). Reverse transcription was then demonstrated via iSciptTM cDNA Synthesis Kit (Bio-Rad, USA) by adding 1 *μ*g of total RNA on CFX96 Touch™ Real-Time PCR Detection System (Bio-Rad, USA). Real-time PCR was performed by means of SsoFast™ EvaGreen® Supermixes (Bio-Rad, USA) on CFX96 Touch™ Real-Time PCR Detection System (Bio-Rad, USA) through adding an equal volume (2 *μ*l) of cDNA products of each sample. The target genes were Slc2a4 (for Glut4; Bio-Rad, qRnoCID0001996, USA) and Acss2 (for AceCS1; Bio-Rad, qRnoCID0005949, USA), *β*-actin was selected as an internal control (forward primer sequence: 5′-CGGTCAGGTCATCACTATC-3′; reverse primer sequence: 5′-TGCCACAGGATTCCATAC-3′). Further ΔCq of the target gene expression was calculated and normalized with *β*-actin by the equation ΔCq= Cq(target) – Cq(*β*-actin).

### 2.8. Statistical Analysis

Values are expressed as means ± SD (standard deviation). Statistical analyses were performed using Prism Software (version 6; GraphPad). Data were not normally distributed based on the Kolmogorov–Smirnov test. Therefore, the cardiac uptakes in the control and HFD groups were compared using Kruskal–Wallis analysis followed by the nonparametric Tukey test. In other experimental data, the comparison of control and HFD groups was done by Student's *t*-test with nonparametric Mann–Whitney test. A *P* value of 0.05 or less was considered statistically significant.

## 3. Results

### 3.1. Physiological and Hemodynamics Remodeling in HFD-Induced IR Hearts

**Figure 2:**
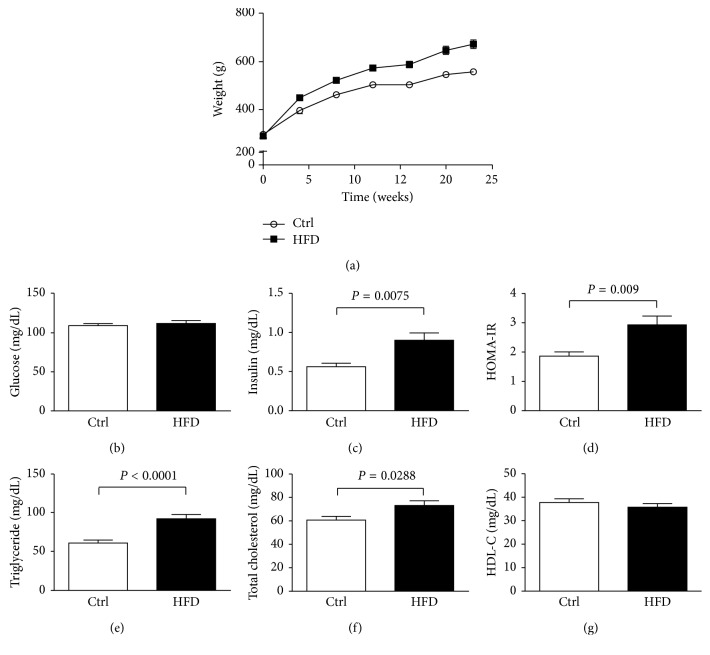
Characterization of HFD-induced obesity model. Rats fed high-fat diet became significantly obese in the time course of body weight (a). Slightly increased glucose concentration in plasma in the HFD group after 10 weeks of high-fat diet feeding (b). Significantly increased insulin (c), HOMA-IR (d), triglyceride (e), and total cholesterol (f) concentration in the HFD group after 10 weeks of high-fat diet feeding. Slightly decreased HDL-C in the HFD group after 10 weeks of high-fat diet feeding (g). Taken together, the HFD rats developed insulin resistance in 10 weeks after high-fat diet feeding. ^*∗*^ *P* < 0.05; ^*∗∗*^ *P* < 0.01; ^*∗∗∗*^ *P* < 0.001 versus control.

After diet alteration for 10 weeks, rats fed HFD developed a phenotype of IR syndrome, which is characterized by hyperlipidemia and hyperinsulinemia and impaired fasting glucose and homeostasis model assessment-insulin resistance (HOMA-IR), as shown in Figure 2. The values of HOMA-IR were obtained by means of the HOMA2 calculator. The body weight, blood insulin concentration, HOMA-IR, triglyceride, and total cholesterol significantly increased in the HFD group (*P* < 0.05), as compared with the control group.

### 3.2. Morphological Effect of HFD on CMR

**Figure 3:**
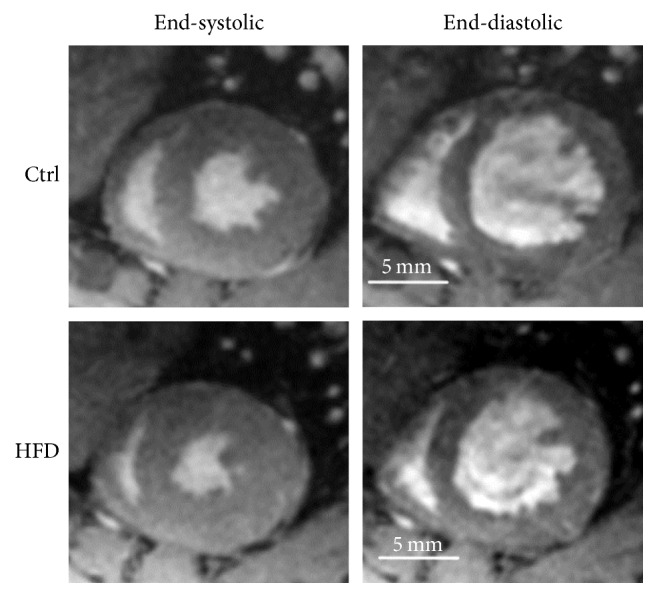
The representative short axis of CMR images in end-systolic and end-diastolic phases. The myocardium CMR images of control and HFD rats in end-systolic and end-diastolic phases are shown. The ejection fraction of the HFD rats significantly increased, and the left ventricle volume of the HFD rats significantly decreased, as compared with control rats based on the QMass analysis.

The representative short axis of CMR images in end-systolic and end-diastolic phases in the control and the HFD rats at 16 weeks of age is demonstrated in Figure 3. *In vivo* cardiac function of control and HFD groups was summarized by CMR with QMass analysis and pool analysis comparison in Table 1. Rats with HFD have a higher ejection fraction, smaller left ventricular end-systolic volume (LVESV), and thicker end-systolic wall thickness than the control group (*P* < 0.05).

### 3.3. Metabolic Effect of HFD on ^18^F-FDG PET

**Figure 4:**
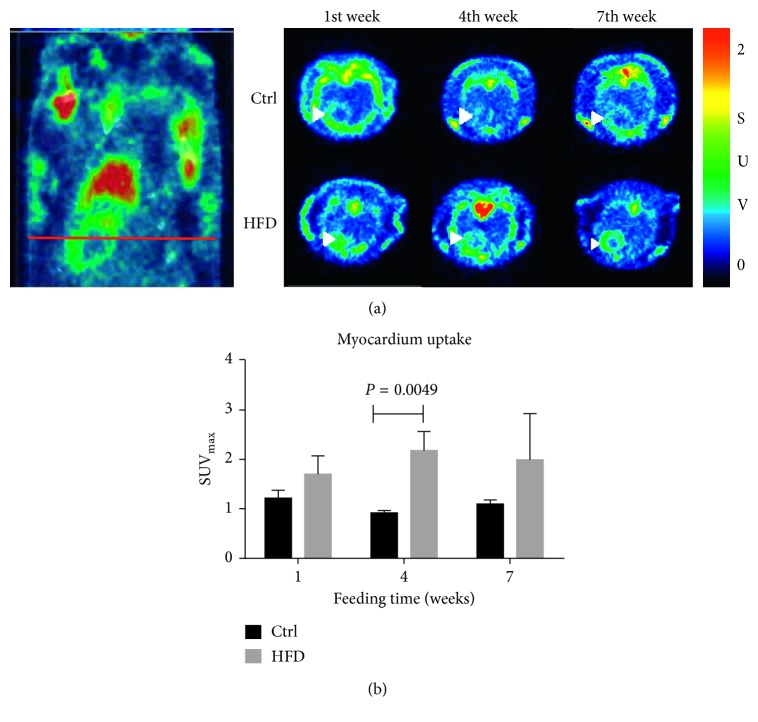
^18^F-FDG PET/CT imaging. (a) Representative midventricular transversal images in a time course. The right image is a corresponding coronal plane ^18^F-FDG PET/CT. (b) Quantitative myocardial glucose uptake by micro-PET after intravenous injection of ^18^F-FDG in the control (*n*=3) and HFD (*n*=3) rats. Significantly increased myocardial ^18^F-FDG uptake in 4 weeks after HFD feeding is noted. ^*∗*^ *P* < 0.05 versus control. Ctrl = control.

HFD rats have altered *in vivo* myocardial ^18^F-FDG uptakes. The SUV_max_ of the left ventricular myocardium was significantly increased in the HFD group than that in the control group over time periods. (SUV_max_, 1.71 ± 0.36 versus 1.22 ± 0.16 at week 1; 2.18 ± 0.39 versus 0.92 ± 0.05 at week 4, *P*=0.0049; 2.00 ± 0.93 versus 1.10 ± 0.08 at week 7), as shown in Figure 4. ^18^F-FDG can be taken up by glycolytic cells and phosphorylated by hexokinase but was trapped in the cells in its phosphorylated form, due to lacks of a 2′ hydroxyl group needed for subsequent metabolism [31]. Therefore, we used [U-^13^C]Glc NMR to further study the downstream metabolism of myocardium glucose utilization.

### 3.4. High-Resolution NMR Analysis of [U-^13^C]Glc Perfused Myocardium

**Figure 5:**
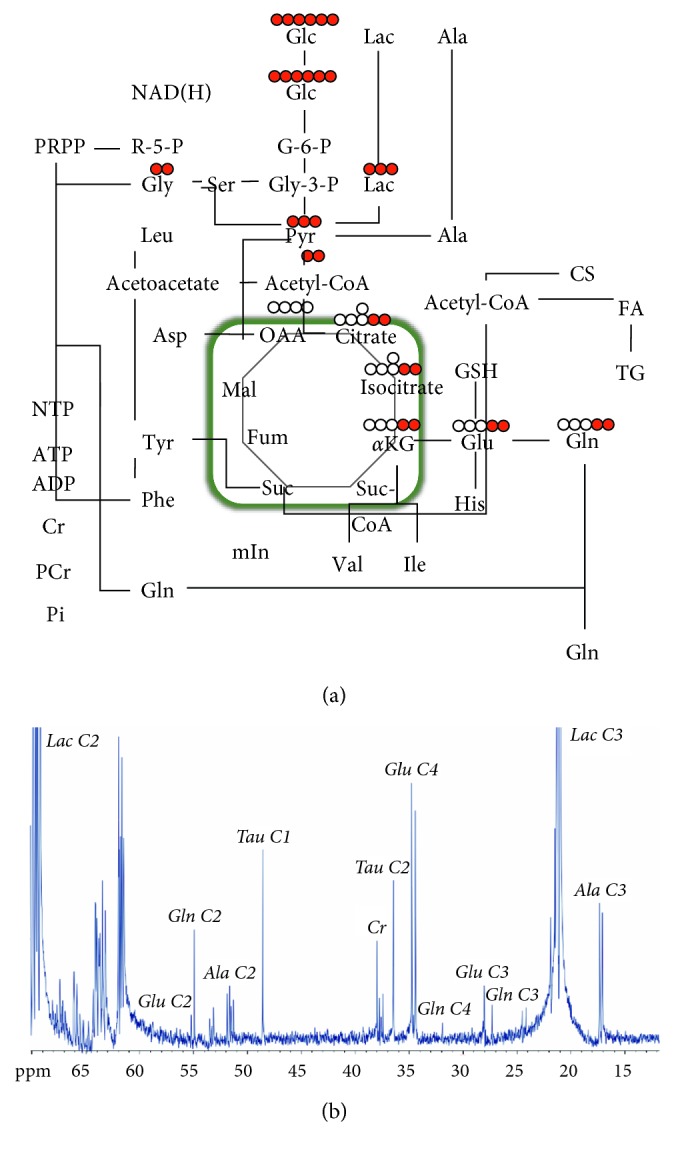
[U-^13^C]-labeled downstream glucose metabolism. (a) The measurement of downstream glucose metabolism by [U-^13^C]-labeled glucose and NMR technique. The carbon atoms of glutamine (Glu45) are from the upstream of glucose metabolism. (b) The ^13^C NMR full spectrum of the myocardium in the HFD rats is demonstrated. After the citric acid cycle, the 4th labeled carbon position in glutamate showed a higher signal than that of the other positions.

There is a list of the steady-state aqueous metabolites associated with the glucose metabolic pathway of the HFD and control rat hearts by ^1^H NMR spectrum analyses in Supplementary Material 1. The majority of myocardial metabolites maintained stable as compared with controls, except levels of acetate, butyrate, glutamine, O-acetylcarnitine, propionate, taurine, and uridine significantly decreased in the HFD groups compared to the control group (*P* < 0.05). Those altered levels of metabolites such as acetate, butyrate, and glutamine were associated with the pathways of glucose metabolism. Other altered levels of metabolites, O-acetylcarnitine, propionate, taurine, and uridine were related to the pathway of fatty acid metabolism. To further interrogate the increased glucose uptake demonstrated on ^18^F-FDG PET, glucose tracers labeled at each carbon position [U-^13^C]Glc was infused into the Langendorff perfusion system, to analyze the glucose utility of the HFD.  Figure 5 reveals the glucose metabolic network identified by the [U-^13^C]Glc NMR system. In myocardium glucose utilization, the ^13^C-carbon of glucose was metabolized to glycine (Gly), lactate, and pyruvate as well as acetyl-CoA. The ^13^C-carbon from acetyl-CoA was contained in citrate, isocitrate, and *α*-ketoglutarate (*α*KG), which synthesizes glutamic acid and glutamine (Gln). The ^13^C-carbon at the 4th and 5th positions of glutamine indicates the utility of [U-^13^C]Glc undergoing oxidative phosphorylation via the TCA cycle. The Glu45/Glu123 ratio of labeled ^13^C-carbon in glucose metabolic substances of HFD groups is significantly higher than that of control groups (18 weeks of age: 0.38 ± 0.03 versus 0.33 ± 0.01; 32 weeks of age: 1.06 ± 0.28 versus 0.39 ± 0.07, *P*=0.017).

### 3.5. Myocardial Overexpression of Glut4, PARP, and LC3B in HFD-Induced IR

**Figure 6:**
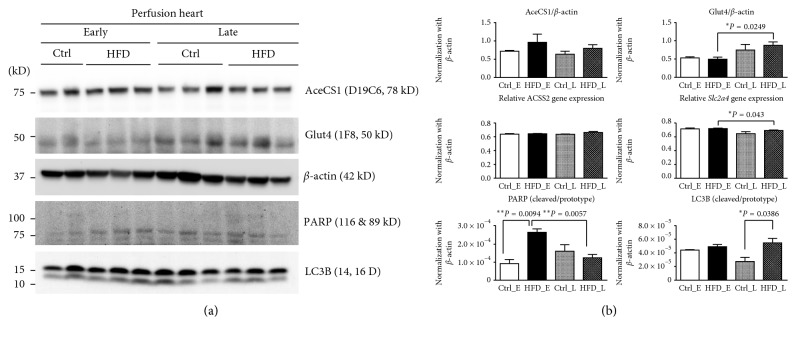
Western blotting and mRNA analysis of myocardium: AceCS1, Glut4, PARP, and LC3B protein expression and *Slc2a4* and *Acss2* gene expression in the perfusion hearts in HFD and control rats in 18 weeks and 36 weeks after high-fat diet feeding. AceCS1 protein expression and *Acss2* gene expression did not show a significant difference between control and HFD in early and late stages. However, mRNA levels change in Glut4, albeit different in trend. Nonetheless, the protein expression of Glut4 increases in accordance with the ^18^F-FDG PET/CT. Decreased cleaved/prototype PARP and increased LC3B-autophagy activation in the myocardium of the HFD rats are noted.

Interestingly, we found the protein expression of Glut4 increased in accordance with the change on the ^18^F-FDG PET (*P* < 0.05). The mRNA levels for Glut4 (*Slc2a4* gene) were also altered (*P* < 0.05). The different trends in mRNA and protein expression might indicate a complex feedback mechanism and warrant further study. The AceCS1 protein and *ACSS2* mRNA expression were not significantly changed during both the early and late HFD feeding. However, in 18 weeks of age rat myocardium, the PARP expression was significantly increased as compared with the control (*P* < 0.05), following to 32 weeks of age, the PARP expression was decreased (early versus late, *P* < 0.05). Notably, the expression of LC3B, an autophagy activation protein, was higher in the 32 weeks of age HFD group compared with the control (*P* < 0.05) (Figure 6).

## 4. Discussion

In the present study, we found the HFD rats in 10 weeks high-fat diet feeding results in mild hyperglycemia, hypercholesterolemia, hyperinsulinemia, and homeostasis model assessment-IR (HOMA-IR), as determined by the biochemical and physiological analysis, with a significant increase in body weight. The significantly increased EF and decreased LVEDV was measured in the HFD rats, indicating that the ten-week HFD fed rats might develop the myocardial hypertrophy, observed as the thicker myocardium wall by CMR. Corresponding to ^18^F-FDG PET study, the myocardium walls of the HFD rats show significant ^18^F-FDG uptake among seven weeks, implying that the HFD rats need amounts of glucose to acquire the energy to support the hypertrophic myocardium activities under glucose utilization in the early stage. Also, with increased glucose metabolite, for example, glutamine and glutamate, and Glut4 expression in late stage, the highly increased myocardium glucose utilization in HFD rats should fully be demonstrated as taking the results together.

We noticed that blood glucose levels between control and high-fat diet groups did not different significantly, which might be attributed to our fasting procedure that is not strict. Even though we removed their food from cages, rats still could eat the bedding materials, fests, and food debris remaining in the cages. Our observations were in line with reports showing no significant difference in glucose levels between control and high-fat diet feeding rats for 9 weeks [32] and 16 weeks [33].

Our results detailed how the Randel cycle replenishes the intermediates of the citric acid cycle. The hemodynamic stress of HFD-induced myocardium overrides fatty acid inhibition of glucose metabolism, which is presumably associated with activation of AMP-activated protein kinase (AMPK), an immediate metabolic adaption and protects the heart from ischemic stress [34]. Interestingly, some previous studies reported that myocardial IR induced by HFD feeding preserves the cardiac contractile function in mild to moderate heart failure rats [35]. Histopathological analysis of the cross-sectional area of myocardium showed significantly increased thickness in HFD rats [24]. We showed that the myocardial hypertrophy induced by HFD needs to consume more energy to support its activity and function by glycose utilization. In line with our results, the increase in myocardial glucose utilization rate compensates the poor fatty acid uptake in the rats with spontaneously hypertension or myocardial infarction [36, 37]. Due to the disturbance in cardiac metabolism, the HFD results in the hyperinsulinemia and hemodynamics remodeling in the pre-T2DM rat model. On the contrary, the cardiomyocytes cross-sectional area increased in the long-term HFD feeding mice [38]. In another mice study with a prolonged HFD for 20 weeks, there was a 40% increase in fatty acid oxidation in the myocardium, whereas glucose oxidation was decreased to 30% of the control [39]. Contrary to our pre-T2DM rat model, the diabetic mouse model appears to be a different metabolic phenotype of cardiopathology.

Some previous reports have demonstrated the alteration of myocardial metabolism in the various diabetic rat models [11]. Shoghi et al. found the decreased myocardial glucose uptake rate in PET images and lower expression of glucose transporters (Glut1 and Glut4) in 20-week-old Zucker Diabetic Fatty (ZDF) rats (fa/fa) but did not show significant difference in morphological changes in the echocardiography measurements [40]. Feeding Wistar rats with high-fat and high-fructose diet for 16 weeks with a small dose of streptozotocin, Menard et al. reported altered myocardium energy metabolism as well as increased myocardial nonesterified fatty acid uptake in the 14 (R, S)-^18^F-fluoro-6-thia-heptadecanoic acid (^18^F-FTHA) micro-PET images [41]. The above evidence supports the metabolic alterations in the myocardium during the development of T2DM.

^18^F-FDG PET demonstrated an early alternation of myocardium glucose utilization in 4-week HFD, whereas the myocardium dysfunction of HFD rats was detected by CMR after 18-week HFD. It seems to be reasonable that the perturbation of myocardium metabolism is followed by the myocardium dysfunction over the progression of the insulin-induced cardiopathological diseases. We have shown the myocardium glucose utilization by *in vivo* glucose analogs PET tracer and ex vivo labeling [U-^13^C]Glc NMR. More recent reports suggested the glutamine as a vital biomarker of metabolic syndromes and insulin-resistant phenotypes [42, 43]. The mice fed oral supplementation of glutamine leading to reduced glucose concentration, indicating glutamine, may play a critical role in the metabolic disease pathways. In our study, we showed that the ratio of labeled C4-5 to C1-3 in glutamine in HFD mice hearts increases, implying either increased Glu_45_ or decreased Glu_123_. Glu_45_ is from [U-^13^C]Glc; Glu_123_ can be from glutamine uptake, which did not occur in our study. The increased glutamine concentration in HFD mice myocardium could be a positive feedback mechanism as the myocardium suffers from the metabolic problem. The further mechanism of glutamine feedback warrants validations in the future. Furthermore, the myocardium fatty acid is considered to predominate the metabolism in the diabetic heart diseases prevailing, which has not been discussed in our study. Even so, several published literature have investigated the myocardium energy utilization by PET radiopharmaceuticals such as carbohydrate metabolism (^18^F-FDG, ^11^C-acetate, and ^11^C-glucose) and fatty acid metabolism (^11^C-palmitate and fatty acid analogs) [44]. The multiple metabolic substrates PET tracers could be our further study direction to investigate the myocardium energy utilization and metabolism.

Cardiac health relies on the heart's ability to utilize different substrates to support overall oxidative metabolism to generate ATP. We demonstrated overexpressed Glut4 and associated changes in mRNA levels in the HFD group, which linked to an increased autophagy activity protein that might intent to generate the additional ATP to support the excessed myocardium activities from the cardiac hypertrophy of HFD rats. The switch from glucose to fatty acid oxidation leads to a less-efficient oxidative phosphorylation because more electrons transported to complex 2 rather than complex 1 in the mitochondrial respiratory chain, hence increasing the production of ROS. Our study represented the early metabolic adaptation of myocardium induced by HFD. The identification of the metabolic phenotypes of chronic diseases including diabetics and heart failure in an early period facilitates the improvement of therapeutic impact [34]. The presence of pre-DM IR associated with a higher reliance on myocardial glucose metabolism. The perturbations in myocardial metabolism may precede an eventual decline of the left ventricular function. Future research should explore the influence of pre-DM status on clinical cardiac ^18^F-FDG PET imaging to better stratify patients for suitable therapeutic intervention.

## 5. Limitations

There are limitations to this study. First, the obese and T2DM heart is less adaptable to myocardial substrate metabolism under anesthesia, known as anesthesia-induced cardioprotection [45]. Anesthesia is unavoidable during in vivo imaging study, and the impact to experimental results still under debate [45, 46]. Therefore, a minimum dose of anesthesia was considered as a concession in our study. Instead of using a continuous dynamic scan, our micro-PET study was acquired 30 min as a static scan, not only keeping conscious during the uptake period but also less time-consuming to adapt clinical situation. For this purpose, we had decided to omit the metabolic rate of glucose with ^18^F-FDG derived from dynamic PET scan and Patlak analysis. Nevertheless, we used a clinical standard quantitative SUV of the myocardium to reflect analog glucose uptake in the regional organs [28].

The glucose utilization is increased in the early HFD-induced myocardial adaptation in the pre-T2DM model, in contrast to the glucose metabolism transit to fatty acid metabolism for energy utility in the myocardium in the late T2DM. Although the preliminary report demonstrated the potential to quantify the myocardial triglyceride content using CMR spectroscopy [47], CMR spectroscopy suffered from the limited sensitivity to detect the glycolysis pathway and was not chosen in this study. ^18^F-FDG, although being transported into the myocardium and phosphorylated by hexokinase as the first step toward glycolysis, is trapped intracellularly as ^18^F-FDG-6-phosphate, thus cannot track down the downstream glycolytic pathway. Therefore, we applied a combination approach of ^18^F-FDG and [U-^13^C]Glc NMR metabolomic analysis trying to understand the whole metabolic landscape. Nevertheless, it is the first time that the downstream metabolic products of pyruvate can be monitored by imaging the augmented form of CMR-hyperpolarized [1-^13^C] pyruvate in real time, and hopefully, these promising results found in this study will have clinical applications in the near future [48].

## 6. Conclusions

In conclusion, HFD-induced IR induces cardiac morphological changes, with a higher ejection fraction, smaller LVESV, and thicker end-systolic wall thickness than the control group. The prediabetic myocardium increased glucose utility undergoing oxidative phosphorylation via the TCA cycle, supported by overexpression of glucose tranporters and acetyl-CoA synthase. Noninvasive imaging biomarker has potentials in detecting the metabolic perturbations prior to the decline of the left ventricular function.

## Figures and Tables

**Table 1:** Comparison of the cardiac function of control and HFD rats.

Cardiac function	Control (*n*=9)	HFD (*n*=9)	*P* value
Ejection fraction (%)	73.32 ± 0.8229	79.50 ± 1.799	0.0096^*∗*^
Stoke volume (*μ*l)	322.2 ± 24.22	317.7 ± 23.52	0.8953
LV volume of ED (*μ*l)	439.9 ± 32.71	399.0 ± 26.79	0.3415
LV volume of ES (*μ*l)	117.7 ± 9.545	81.33 ± 7.833	0.0081^*∗∗*^
Cardiac output (*μ*l/min)	133.7 ± 10.06	131.9 ± 9.765	0.8987
ED segmented wall thickness (mm)	3.061 ± 0.068	3.391 ± 0.098	0.0168^*∗*^

Values are expressed as mean ± SD. The cardiac function of the control and HFD rats was measured from CMR images and QMass Software; ^*∗*^ *P* < 0.05 and ^*∗∗*^ *P* < 0.01 versus control; LV = left ventricle; ED = end-diastolic volume; ES = end-systolic volume.

## Data Availability

The NMR, western blot, and mRNA data used to support the findings of this study are available from the corresponding author upon reasonable request. The CMR and PET scan images data used to support the findings of this study are available from Yi-Hsiu Chung upon reasonable request.

## Abbreviations

HFD: High-fat diet
T2DM: Type 2 diabetes mellitus
CMR: Cardiac magnetic resonance
^18^F-FDG: ^18^F-fluorodeoxyglucose
PET: Positron emission tomography
NMR: Nuclear magnetic resonance
[U-^13^C]Glc: Carbon-13-labeled glucose
HOMA-IR: Homeostasis model assessment-IR
IR: Insulin resistance
SUV_max_: Maximum standardized uptake values.

## Supplementary Materials

Supplementary Materials Supplementary Material 1: the majority of myocardial metabolites maintained stable as compared with controls, except levels of acetate, butyrate, glutamine, and O-acetylcarnitine.
