# Supplementary material for: Early Imaging Biomarker of Myocardial Glucose Adaptations in High-Fat-Diet-Induced Insulin Resistance Model by Using 18F-FDG PET and [U-13C]glucose Nuclear Magnetic Resonance Tracer
Source: Contrast Media Mol Imaging. 2018 Jul 12;2018:8751267. doi: 10.1155/2018/8751267 (PMC6079607; doi:10.1155/2018/8751267)
Supplement: Supplementary Materials — Supplementary Material 1: the majority of myocardial metabolites maintained stable as compared with controls, except levels of acetate, butyrate, glutamine, and O-acetylcarnitine. [file 8751267.f1.docx]

**Supplemental data 1 Table 1 Metabolite concentration (μM/mg) and fold change (HFD/Control) of fresh heart**

|  | Control (n=2) | HFD (n=3) | Fold change (HFD/Control) | *P* value |  |
| --- | --- | --- | --- | --- | --- |
| 3-Hydroxybutyrate | 0.0172 | 0.0093 | 0.5389 | 0.1386 |  |
| ADP | 0.0051 | 0.0049 | 0.9602 | 0.8230 |  |
| AMP | 0.0080 | 0.0082 | 1.0264 | 0.9399 |  |
| ATP | 0.0218 | 0.0229 | 1.0500 | 0.8140 |  |
| Acetate | 0.2642 | 0.1565 | 0.5924 | 0.0284 | * |
| Acetone | 0.0019 | 0.0013 | 0.6565 | 0.0359 | * |
| Alanine | 0.1423 | 0.1589 | 1.1167 | 0.6985 |  |
| Arginine | 0.0445 | 0.0398 | 0.8951 | 0.4510 |  |
| Asparagine | 0.0357 | 0.0259 | 0.7257 | 0.1103 |  |
| Aspartate | 0.0809 | 0.0849 | 1.0491 | 0.8503 |  |
| Butyrate | 0.0095 | 0.0054 | 0.5673 | 0.0096 | ** |
| Carnitine | 0.0466 | 0.0341 | 0.7307 | 0.2968 |  |
| Choline | 0.0092 | 0.0073 | 0.7879 | 0.3073 |  |
| Creatine | 1.2198 | 0.9230 | 0.7567 | 0.1245 |  |
| Creatine phosphate | 0.0078 | 0.0099 | 1.2591 | 0.5632 |  |
| Creatinine | 0.0230 | 0.0163 | 0.7098 | 0.1546 |  |
| Cytidine | 0.0063 | 0.0059 | 0.9329 | 0.6829 |  |
| Ethylene glycol | 0.0106 | 0.0083 | 0.7782 | 0.2287 |  |
| Formate | 0.1029 | 0.0712 | 0.6914 | 0.1358 |  |
| Fructose | 0.0583 | 0.0450 | 0.7731 | 0.6047 |  |
| Fumarate | 0.0182 | 0.0231 | 1.2707 | 0.1605 |  |
| Glucose | 0.0859 | 0.0699 | 0.8143 | 0.7077 |  |
| Glucose-1-phosphate | 0.0067 | 0.0031 | 0.4612 | 0.4095 |  |
| Glucose-6-phosphate | 0.0709 | 0.0403 | 0.5687 | 0.5474 |  |
| Glutamate | 0.1912 | 0.2661 | 1.3920 | 0.1240 |  |
| Glutamine | 0.4037 | 0.2118 | 0.5246 | 0.0046 | ** |
| Glutathione | 0.0293 | 0.0279 | 0.9530 | 0.8180 |  |
| Glycerol | 0.0736 | 0.0753 | 1.0239 | 0.9318 |  |
| Glycine | 0.0342 | 0.0315 | 0.9237 | 0.7035 |  |
| Guanosine | 0.0105 | 0.0089 | 0.8444 | 0.1948 |  |
| Histidine | 0.0130 | 0.0105 | 0.8068 | 0.0934 |  |
| IMP | 0.0023 | 0.0051 | 2.2045 | 0.2268 |  |
| Inosine | 0.2652 | 0.2127 | 0.8023 | 0.1431 |  |
| Isoleucine | 0.0116 | 0.0103 | 0.8868 | 0.5514 |  |
| Lactate | 1.1458 | 0.8708 | 0.7600 | 0.4089 |  |
| Lactose | 0.0473 | 0.0193 | 0.4079 | 0.2770 |  |
| Leucine | 0.0232 | 0.0184 | 0.7935 | 0.2262 |  |
| Lysine | 0.0431 | 0.0389 | 0.9010 | 0.4566 |  |
| Malate | 0.0521 | 0.0667 | 1.2801 | 0.3336 |  |
| Mannose | 0.0282 | 0.0143 | 0.5060 | 0.3746 |  |
| Methanol | 0.0540 | 0.0367 | 0.6793 | 0.0887 |  |
| Methionine | 0.0251 | 0.0190 | 0.7565 | 0.1187 |  |
| NAD+ | 0.0035 | 0.0030 | 0.8701 | 0.5156 |  |
| NADH | 0.0025 | 0.0054 | 2.1996 | 0.0009 | *** |
| Niacinamide | 0.0511 | 0.0444 | 0.8679 | 0.1687 |  |
| O-Acetylcarnitine | 0.0333 | 0.0170 | 0.5107 | 0.0040 | ** |
| O-Phosphocholine | 0.0306 | 0.0213 | 0.6959 | 0.0686 |  |
| Phenylalanine | 0.0098 | 0.0078 | 0.7898 | 0.0809 |  |
| Proline | 0.0387 | 0.0396 | 1.0239 | 0.9161 |  |
| Propionate | 0.0065 | 0.0040 | 0.6086 | 0.0403 | * |
| Serine | 0.0414 | 0.0306 | 0.7384 | 0.3594 |  |
| Succinate | 0.0077 | 0.0019 | 0.2438 | 0.1459 |  |
| Sucrose | 0.0075 | 0.0045 | 0.5981 | 0.4168 |  |
| Taurine | 2.6295 | 2.0439 | 0.7773 | 0.0333 | * |
| Threonine | 0.0591 | 0.0509 | 0.8618 | 0.1964 |  |
| Tryptophan | 0.0042 | 0.0038 | 0.9100 | 0.4771 |  |
| Tyrosine | 0.0112 | 0.0084 | 0.7511 | 0.0735 |  |
| Uracil | 0.0065 | 0.0057 | 0.8825 | 0.3984 |  |
| Uridine | 0.0060 | 0.0038 | 0.6322 | 0.0380 | * |
| Valine | 0.0173 | 0.0161 | 0.9305 | 0.6252 |  |
| myo-Inositol | 0.0388 | 0.0293 | 0.7558 | 0.1474 |  |
| sn-Glycero-3-phosphocholine | 0.0341 | 0.0281 | 0.8223 | 0.1128 |  |
| π-Methylhistidine | 0.0049 | 0.0068 | 1.3922 | 0.0793 |  |
